# External Limiting Membrane Disruption Predicts Long-Term Outcome in Strict Treat-And-Extend Regimen in Neovascular Age-Related Macular Degeneration

**DOI:** 10.3389/fmed.2021.706084

**Published:** 2021-09-03

**Authors:** Laura Hoffmann, Katja Hatz

**Affiliations:** ^1^Department of Ophthalmology, Vista Augenklinik Binningen, Binningen, Switzerland; ^2^Faculty of Medicine, University of Basel, Basel, Switzerland

**Keywords:** AMD, predictor, treat-and-extend, external limiting membrane, anti-VEGF, long-term

## Abstract

The use of anti-vascular-endothelial growth factor agents for neovascular age-related macular degeneration (nAMD) in different treatment schemes is widely common in clinical practice. However, there is currently limited data on the long-term outcomes of a strict treat-and-extend regimen (TER) and imaging biomarkers to predict both functional outcome and the potential for a TER exit due to success. In this retrospective study we followed treatment-naïve subjects with nAMD starting treatment with either ranibizumab or aflibercept in a TER without loading dose but with predefined exit criteria for up to 8 years. We evaluated both the functional outcome and several spectral-domain optical coherence tomography parameters in a follow-up mode using a standardized protocol. Within the 211 eyes followed for a mean of 60.3 ± 20.9 months, follow-up adherence was high with major part of discontinuations of TER being due to success. Mean best-corrected visual acuity (BCVA) increased from initially 63.9 ± 15.5 ETDRS letters to 70.0 ± 14.7 after 1 year (+6.1 letters, *p* < 0.001) and to 68.5 ± 18.1 (+4.6 letters, *p* = 0.028) at 5 years. A worse BCVA (*p* = 0.001) and a better external limiting membrane (ELM) disruption score at baseline predicted (*p* = 0.019) BCVA gain at 5 years. The probability of reaching the exit criteria was significantly associated with a better ELM disruption score (*p* = 0.044) and the absence of a central pigment epithelial detachment (PED) (*p* = 0.05) at baseline. Significant visual gains were sustained in a long-term TER in a real-world setting. Integrity of ELM at baseline predicted BCVA gain at 5 years and the potential for TER exit due to success.

## Introduction

Nowadays, intravitreal injection of anti-vascular endothelial growth factor (VEGF) agents is acknowledged as the standard treatment in neovascular age-related macular degeneration (nAMD). Whereas, unprecedented visual gains were achieved in the pivotal studies for ranibizumab (1) and aflibercept (2) with monthly respectively bimonthly intravitreal injections (IVT), the high associated treatment burden claimed the development of individualized dosing protocols. Accordingly, the pro re nata (PRN) and treat-and-extend regimen (TER) achieved frequent use in clinical practice. Compared to the PRN schedule with an IVT “as-needed” based on monthly visits, the TER consists in a proactive approach with a progressive extension of treatment and visit intervals guided by activity criteria based on optical coherence tomography (OCT) findings and biomicroscopic examination according to predefined stability criteria. The use of pre-established exit criteria permitting the treatment cessation upon disease stability may be useful to prevent unnecessary continuation of injections (3). Better visual outcomes with fewer clinic visits were reported in nAMD patients treated with ranibizumab in a TER compared to a PRN schedule for up to 3 years (4, 5).

However, translation of the results achieved in the highly controlled landmark trials for anti-VEGF therapy applying stringent inclusion criteria to routine clinical practice remains difficult (6). Recent studies report the maintenance of stable visual acuity using a TER with aflibercept under real-world conditions in patients followed for up to 4 years (7, 8). Meanwhile, there is currently limited data on the long-term outcomes of TER allowing a switch between the different agents whereby possibly including more poor responders (9, 10). Previously, visual acuity after resolution of exsudation by intravitreal anti-VEGF treatment has been correlated with the status of the photoreceptor integrity on OCT (11). Further, early poor responders presented with a loss of the external limiting membrane (ELM) at baseline (12). However, in order to handle patients' expectations and adapt treatment strategies, baseline predicting factors resulting in a long-term functional and anatomic treatment response need to be identified. Therefore, this study aims to evaluate the efficacy and outcome predicting factors in intravitreal anti-VEGF therapy following a TER with exit strategy in treatment-naïve patients with nAMD for up to 8 years.

## Methods

This was a retrospective single-center study which evaluated anti-VEGF treatment between 2012 and 2020 at Vista Klinik Binningen, Switzerland. Approval to conduct this study was obtained from the local ethics committee [Ethikkommission Nordwestschweiz (EKNZ No BASEC 2020-01294)], and the study was performed in accordance with ICH-GCP guidelines and followed the tenets of the Declaration of Helsinki. According to local requirements general informed consent regarding retrospective analyses of data and use of imaging material was obtained from the patients. Eligible were treatment-naïve patients with newly fluorescein angiography diagnosed macular neovascularization (MNV) of all subtypes due to nAMD starting intravitreal anti-VEGF therapy in a treat-and-extend regimen (TER) using either aflibercept 2 mg (Eylea; Bayer, Switzerland) or ranibizumab 0.5 mg (Lucentis; Novartis AG, Basel, Switzerland). Excluded were subjects with baseline corrected visual acuity <20/200 (Snellen) or any other previous or adjunct treatment for nAMD including anti-VEGF therapy in a PRN schedule, stereotactic radiotherapy or photodynamic therapy or a follow-up of <18 months.

Within this clinical routine setting a standard TER without loading dose but with exit strategy was used (13). Beside visual acuity assessment, biomicroscopic examination and Spectral-domain Optical Coherence Tomography (SD-OCT) imaging, each visit included an intravitreal injection and beginning from visit 2 (week 4) onwards the TER interval was either extended or shortened by 2 (up to 2014 by 4) weeks (minimum of 4 weeks) depending on predefined criteria: If no signs of intra- or subretinal fluid were observed on SD-OCT or had remained stable for three consecutive visits and no new hemorrhage was visible, treatment intervals were extended; in the case of instability (new or increased sub- or intraretinal fluid compared to the last two previous visits and the “dryest” SD-OCT during follow-up or presence of a new hemorrhage) intervals were shortened. If the maximum interval of 12 weeks (from 2016 onwards 14 weeks) was maintained three consecutive times without recurrence, a further visit with the option of TER exit in the case of persisting MNV inactivity was performed 14 weeks (from 2016 onwards 16 weeks) after the last injection, followed by at least 2-monthly follow-up visits. After an exit of TER due to success, the time span between the last injection and an eventual recurrence was recorded.

**Figure 1:**
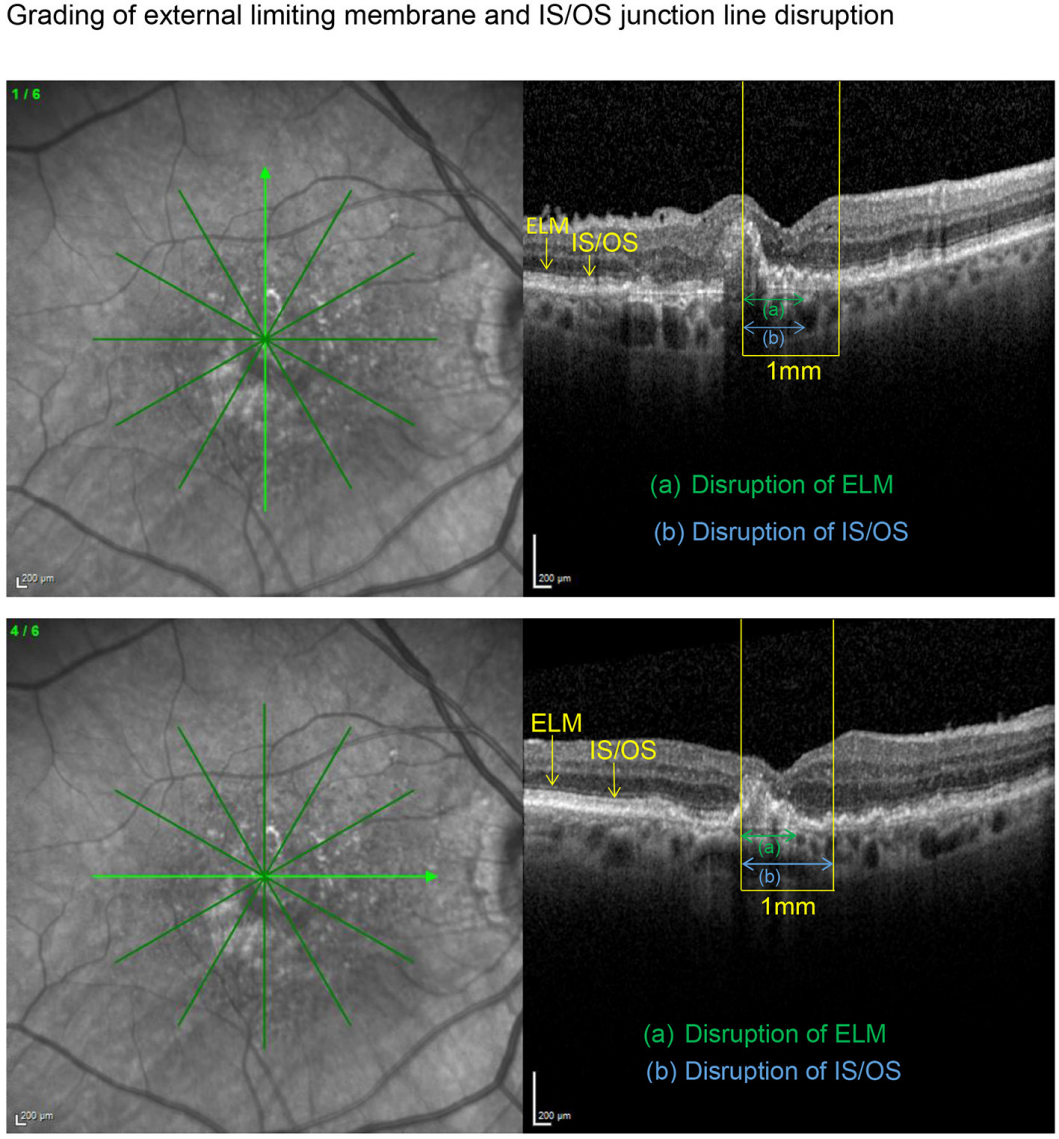
Vertical and horizontal OCT scans in the macular star setting with an ETDRS grid overlay centered on the fovea. After identification of the central 1 mm (yellow), the length of the disrupted external limiting membrane (ELM) (green) and IS/OS junction line (blue) were measured with the measuring tool. Grading was carried out as described in the methods section. Subsequently, both disruption scores were calculated as the means of the corresponding scores obtained in the horizontal and vertical scan, respectively. In this example resulted an ELM disruption score of 2 and an IS/OS disruption score of 2.5 with an impaired BCVA of 58 letters.

**Figure 2:**
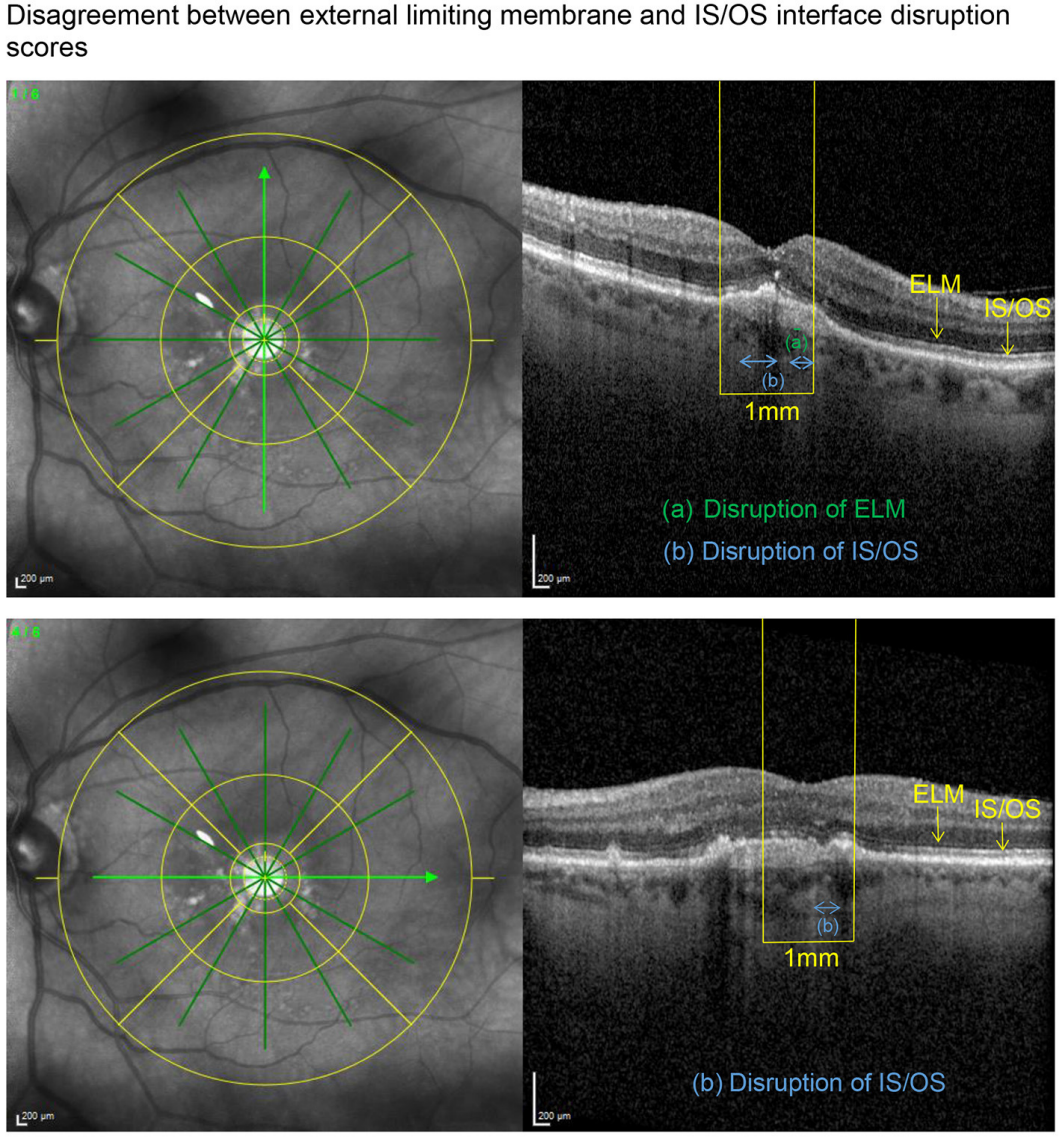
Applying the grading system to an eye with a preserved BCVA of 80 letters, this example illustrates an only mildly disrupted ELM (impairment score of 0.5) in the presence of an already altered IS/OS interface (disruption score of 1.5).

SD-OCT scans were acquired using an established protocol consisting of volume scans. For the volume scan of 20° × 15°, 19 frames (High Speed mode, 9 frames, 512 A-scans) were acquired in a follow-up setting. Further, a 6-mm star scan (High Speed mode, 9 frames, 512 A-scans) was performed. Quantitative and qualitative evaluations were performed by one physician (LH) according to a standardized protocol. Photoreceptor inner segment and outer segments (IS/OS) interface and external limiting membrane (ELM) impairment were evaluated in the macular star scan by calculating the mean of the disruption scores of the horizontal and vertical scans. Impairment of ELM and IS/OS in the central 1 mm were graded from 0 (intact) to 3 (severe) as previously described in diabetic macular oedema (14, 15). The following grading was used: 0 (no disruption in 1-mm center), 1 (mild disruption <1/4 within 1-mm center), 2 (1/4–3/4 disruption within 1-mm center), and 3 (>3/4 disruption within 1-mm center), see Figures 1, 2. Geographical atrophy was assessed by fundus autofluorescence. BCVA values were converted to their Early Treatment Diabetic Retinopathy Study (ETDRS) letter score equivalents (16).

Statistical analyses were performed with SPSS version 21 (SPSS, Inc., Chicago, IL). Descriptive data were presented as mean ± standard deviation (SD) or percentages. Paired and unpaired *t*-tests were performed to compare baseline with follow-up values or differences between subgroups. Pearson correlation coefficients were calculated for correlation analyses. Significant predictors of functional and anatomic outcomes in univariate analysis were subsequently entered in multivariate regression analyses (linear and logistic models) with a backward selection procedure. *P* < 0.05 were considered statistically significant.

## Results

Two hundred-eleven eyes from 187 patients were included. Mean patient age was 77.9 ± 8.0 years with 116 females (62%) and 71 males (38%). Baseline characteristics are summarized in Table 1. The mean length of total follow-up was 60.3 ± 20.9 months (range 18–106) with a mean follow-up under TE regimen of 53.7 ± 23.3 months (range 18–106). While all eyes had a follow-up of ≥18 months, 198 were followed up for ≥2 years, 185 for ≥3 years, 164 for ≥4 years, 134 for ≥5 years, 71 for ≥6 years, 35 for ≥7 years and 13 for up to 8 years. 112 eyes (53.1%) discontinued TER in our clinic during the observation period whereof 64 (57.1%) due to success (regular TER exit, 30.3% of all eyes), 21 (18.8%) deceased between start of treatment and 2020, 17 (15.2%) at their own request including cases lacking functional potential and 9 (8.0%) due to relocation. While all patients started TER with either ranibizumab or aflibercept, 126 eyes (59.7%) switched treatment agents during follow-up, on average 1.13 times (Table 2). The mean time span from baseline to the first switch and from the previous switch to the second and third switch were 14.5 ± 12.0, 43.5 ± 19.1, and 10.0 ± 7.1 months, respectively. The proportion of eyes treated with aflibercept increased from 10.4% at baseline to 80% at year 6 (Table 2).

**Table 1:** Baseline characteristics.

**Characteristic (*n* = 211)**	
Age at baseline, years ±SD	77.9 ± 8.0
Female sex, *n* (%)	116 (62.0)
Mean BCVA, ETDRS letters ±SD (Snellen)	63.8 ± 15.5 (20/55)
**Lesion type**	
Type 1 MNV, *n* (%)	127 (60.2)
Mixed type 1 and 2 MNV, *n* (%)	31 (14.7)
Type 2 MNV, *n* (%)	43 (20.4)
Type 3 MNV, *n* (%)	10 (4.7)
Presence of CNV in central 1 mm, *n* (%)	146 (69.2)
**Location of fluid**	
Intraretinal only, *n* (%)	48 (22.7)
Subretinal only, *n* (%)	121 (57.3)
Intraretinal and subretinal, *n* (%)	42 (19.9)
Mean CRT±SD, μm	405.4 ± 122.5
Presence of central geographic atrophy, *n* (%)	54 (25.6)
Presence of pigment epithelial detachment in central 1 mm, *n* (%)	152 (72.4)

**Table 2:** Distribution of anti-VEGF agents.

	**1st year**	**2nd year**	**3rd year**	**4th year**	**5th year**	**6th year**
**Anti-VEGF agent received**						
Ranibizumab, *n* (%)	189 (89.6)	107 (50.5)	62 (33.5)	41 (25.7)	29 (22.0)	20 (20.0)
Aflibercept, *n* (%)	22 (10.4)	104 (49.5)	123 (66.5)	118 (74.3)	103 (78.0)	80 (80.0)
Number of eyes being switched	82	18	12	6	5	3

### Functional Outcomes

**Figure 3:**
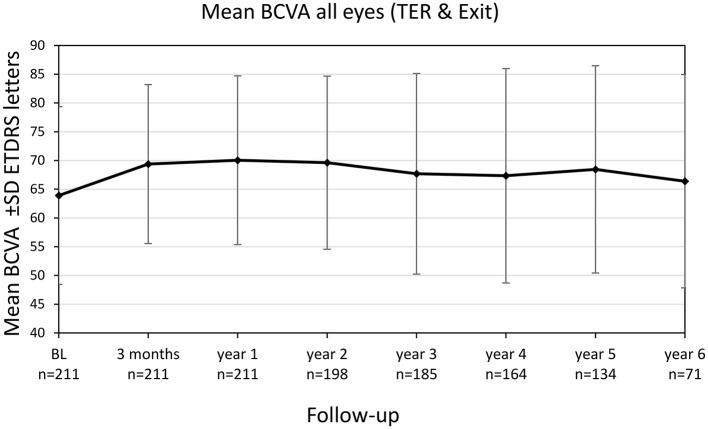
Best-corrected visual acuity (BCVA) change of all eyes (treat-and-extend regimen & exit) from baseline until year 6. Values are shown in Early Treatment of Diabetic Retinopathy Study (ETDRS) letters ± SD, standard deviation.

Mean BCVA for all eyes (still in TER and eyes after exit of TER) improved significantly from 63.9 ± 15.5 letters (20/55) at baseline to 69.4 ± 13.9 letters (20/40) after 3 months (+5.5 letters, *p* < 0.001) and to 70.0 ± 14.7 (20/40) letters after 1 year (+6.1 letters, *p* < 0.001), see Figure 3. The improvement in BCVA was significant until the fifth year of treatment [68.5 ± 18.1 letters (20/43), +4.6 letters, *p* = 0.028]. Thereafter, BCVA still remained stable compared to baseline (+2.5 letters at year 6, *p* = 0.774).

Analysis including only the eyes still treated within TER revealed comparable BCVA gains with a significant increase from 63.9 ± 15.5 letters (20/55) at baseline to 69.4 letters (20/40) after 3 years (+5.5 letters, *p* < 0.001). The gain in BCVA remained significant until year 6 of treatment (+6.2 letters, *p* = 0.007) and subsequently BCVA gains stabilized compared to baseline.

Out of all eyes (TER and exit) 16 eyes had suffered a vision loss ≥15 letters at year five whereof 10 due to geographical atrophy, 5 due to sub-retinal fibrosis and one caused by a macular hemorrhage. The proportion of eyes reaching a vision gain ≥15 letters remained stable throughout the observation period with 22.4% of eyes at year five (Table 2). No significant differences regarding BCVA gain after 5 years of treatment could be shown between eyes treated continuously with either ranibizumab (*n* = 65) or aflibercept (*n* = 20) during the entire observation period (*p* = 0.899).

### Injection Frequencies and Treatment Intervals

**Figure 4:**
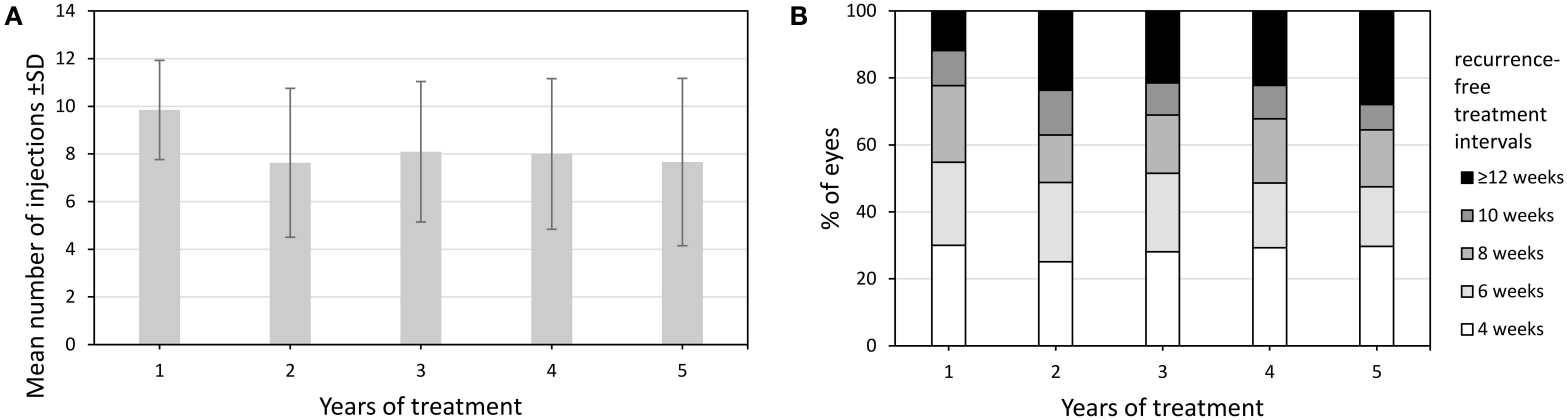
**(A)** Number of injections of eyes treated in TER from baseline to year 5. **(B)** Change in distribution of recurrence-free treatment intervals of eyes treated in TER from year 1 to year 5.Values are shown as mean ± SD, standard deviation and percentages.

Within the TER eyes received a total of 35.6 ± 20.8 injections (mean ± SD) during follow-up. Most of them were administered during the first year and significantly less during the second year (9.9 ± 2.1 vs. 7.4 ± 3.3, *p* < 0.001) (Figure 4). Subsequently, the number of injections remained stable throughout the observation period. The mean maximum recurrence-free treatment interval (RFTI) increased from 7.1 ± 2.6 weeks during the first year to 8.5 ± 3.9 weeks after 5 years (*p* < 0.001). Likewise, the mean maximum treatment interval could be extended from 8.1 ± 2.5 weeks during the first year to 9.0 ± 3.9 weeks in the fifth year (*p* < 0.001). While 11.9% of the eyes reached the maximum RFTI of 12 weeks after the first year, this proportion could be extended to 27.9% after 5 years, see Figure 4. Sixty-four eyes (30.3%) reached the exit from TER whereof 51 (79.7%) in the first 5 years. Twenty-two eyes (34.4%) experienced a recurrence occurring after a mean of 11.2 ± 6.7 months.

### Morphologic Characteristics

**Figure 5:**
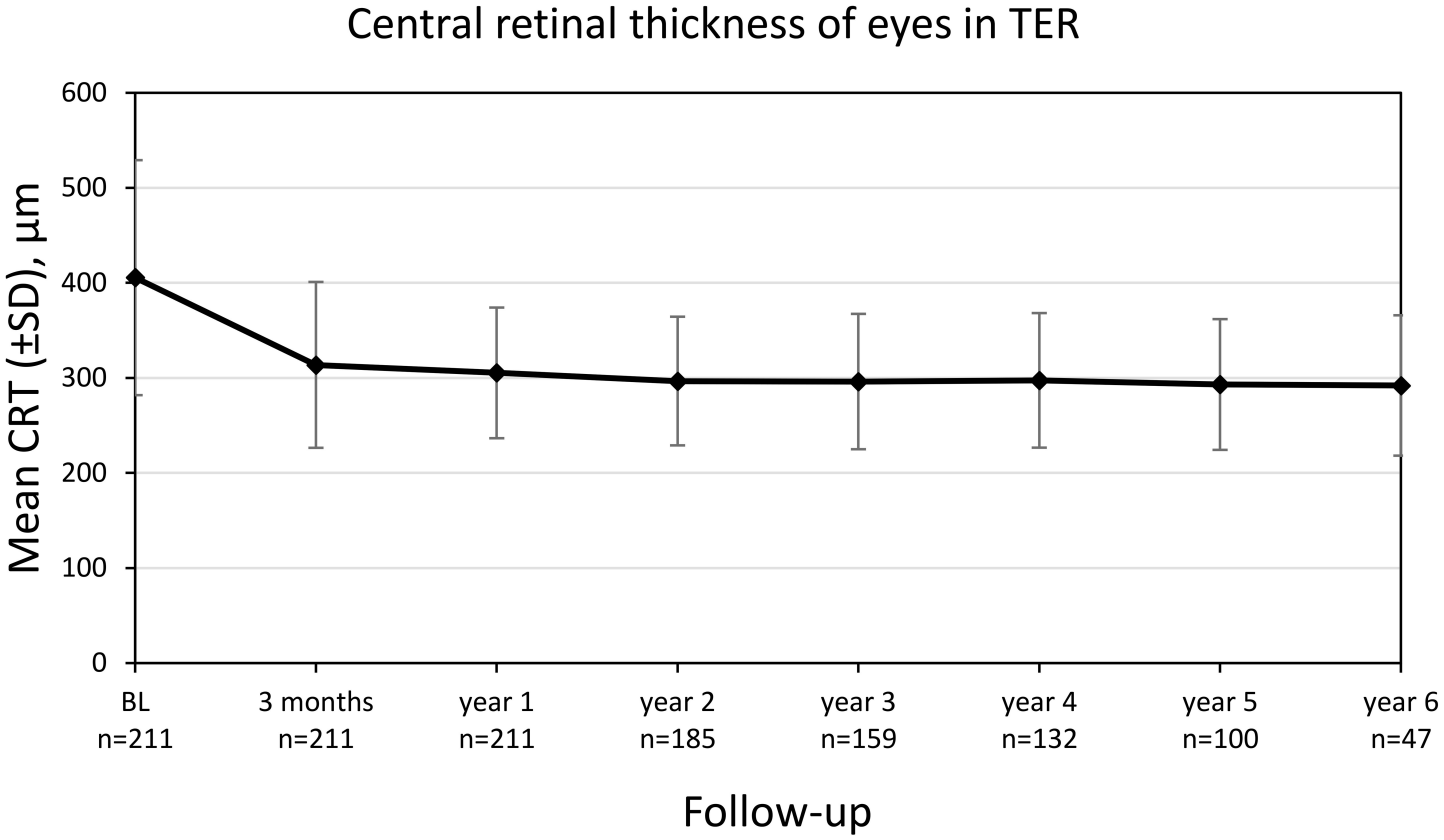
Central retinal thickness (CRT) of eyes treated in TER from baseline to year 6. Values are shown as mean ± SD, standard deviation.

The central retinal thickness (CRT) decreased significantly from 405.4 ± 122.5 μm at diagnosis to 313.8 ± 87.3 μm after 3 months (*p* < 0.001) with a consecutive stabilization (Figure 5). There was a weak negative correlation between CRT and BCVA at baseline (*r* = −0.350, *p* < 0.001). The proportion of eyes reaching dry macular conditions increased to 55.7% after 2 years and remained stable until the end of observation (Table 3).

**Table 3:** Change in functional and morphological outcomes during follow-up.

**All eyes (TER & follow-up after exit)**	**Baseline**	**1st year**	**2nd year**	**3rd year**	**4th year**	**5th year**	**6th year**
	***n* = 211**	***n* = 211**	***n* = 198**	***n* = 185**	***n* = 164**	***n* = 134**	***n* = 71**
Mean BCVA letters ETDRS ±SD (Snellen)	63.9 ± 15.5 (20/55)	70.0 ± 14.7 (20/40)	69.6 ± 15.1 (20/40)	67.7 ± 17.5 (20/44)	67.3 ± 18.8 (20/45)	68.5 ± 18.1 (20/43)	66.4 ± 18.8 (20/47)
Loss ≥15 letters ETDRS, *n* (%)	/	11 (5.2)	13 (6.6)	21 (11.4)	20 (12.2)	16 (11.9)	12 (16.9)
Gain ≥15 letters ETDRS, *n* (%)	/	54 (25.7)	50 (25.3)	42 (22.7)	40 (24.4)	30 (22.4)	11 (15.5)
**Eyes in TER**	**Baseline**	**1st year**	**2nd year**	**3rd year**	**4th year**	**5th year**	**6th year**
	***n*** **=** **211**	***n*** **=** **211**	***n*** **=** **185**	***n*** **=** **159**	***n*** **=** **132**	***n*** **=** **100**	***n*** **=** **47**
Mean BCVA letters ETDRS ±SD (Snellen)	63.9 ± 15.5 (20/55)	70.0 ± 14.7 (20/40)	70.3 ± 14.6 (20/40)	69.4 ± 15.8 (20/41)	68.6 ± 17.9 (20/43)	71.1 ± 15.5 (20/38)	70.1 ± 13.4 (20/40)
**Location of fluid**							
Dry, *n* (%)	0	98 (46.4)	103 (55.7)	76 (48.3)	71 (53.8)	48 (48.0)	21 (44.7)
Intraretinal, *n* (%)	48 (22.7)	42 (19.9)	31 (16.8)	37 (21.8)	29 (22.0)	24 (24.0)	12 (25.5)
Subretinal, *n* (%)	121 (57.3)	60 (28.4)	44 (23.8)	43 (27.0)	29 (22.0)	26 (26.0)	11 (23.4)
Intraretinal and subretinal, *n* (%)	42 (19.9)	11 (5.2)	5 (2.7)	3 (1.9)	1 (0.8)	2 (2.0)	3 (6.4)
Mean IS/OS interface disruption score	2.19	1.74	1.72	1.72	1.71	1.69	1.66
Mean ELM disruption score	1.83	1.35	1.35	1.33	1.32	1.21	1.26
Presence of PED in the central 1 mm, *n* (%)	152 (72.4)	141 (66.8)	124 (67.0)	110 (69.2)	93 (70.5)	68 (68.0)	35 (74.5)

The IS/OS interface and ELM disruption scores decreased significantly after 1 year of treatment and stabilized thereafter (2.2 ± 0.9 vs. 1.74 ± 1.0 and 1.8 ± 1.0 vs. 1.4 ± 1.0, *p* < 0.001, respectively) (Table 3). Both disruption scores were highly correlated with BCVA during the whole observation period. Namely, correlation coefficients between IS/OS disruption score and BCVA were −0.555 at baseline, −0.617 at year 1 and −0.604 after 5 years (*p* < 0.001, respectively). Likewise, ELM disruption score was strongly correlated with BCVA at diagnosis (*r* = −0.553), after 1 year (*r* = −0.693) and after 5 years (*r* = −0.710) (*p* < 0001, respectively).

### Outcome Predictors

In univariate analysis, while the presence of a MNV in the central 1 mm at baseline was associated with a significantly worse BCVA during the first 5 years of treatment (*p* < 0.001), these eyes showed significantly higher BCVA gains than eyes without central MNV during year 1 and 2 (+7.6 letters vs. +3.4 letters, *p* = 0.004 and +6.9 letters vs. 3.0 letters, *p* = 0.022). While type 2/mixed type 1–2 lesions (*n* = 74) had significantly worse baseline BCVA than type1/type3 lesions (*n* = 137) (*p* = 0.023), eyes with type 2 MNV (*n* = 43) showed significantly higher BCVA gain after the first and fifth year compared to the other lesion types (+13.0 vs. +4.6 letters, *p* < 0.001 and +8.8 vs. +2.3 letters, *p* = 0.044). Subsequent multivariate regression analysis identified younger age (*p* = 0.049), a worse baseline BCVA (*p* < 0.001) and the absence of a central PED (*p* = 0.046) as significant predictors for a higher BCVA gain at year 1 whereas a better BCVA score at year 1 was only significantly associated with a higher baseline BCVA (*p* < 0.001). After 5 years of follow-up, significant predictors of BCVA gain and BCVA score were the baseline BCVA (*p* = 0.001), a better ELM disruption score at baseline (*p* = 0.019) and the absence of central GA at baseline (*p* = 0.006). Furthermore, logistic regression analysis including several morphologic parameters selected a low CRT (*p* = 0.035), a better ELM disruption score (*p* = 0.044) and the absence of a central PED at baseline (*p* = 0.05) as independent variables predicting early disease control permitting the exit of the TER in the first 5 years.

## Discussion

Our study is the first to report a significant visual gain of almost one line ETDRS chart after 5 years using a strict TER with exit-strategy in a clinical setting (7–9, 17). We included a bigger cohort compared to previously published long-term studies [211 vs. 82 (8) and 101 (10) eyes, respectively] with a greater treatment adherence [88 vs. 48% (7), respectively, 74% (8) at 3 years, 78% vs. 27% (7), respectively, 61% (8) at 4 years and 64% vs. 40% (9), respectively, 25% (18) at 5 years]. Furthermore, the inclusion of a population treated with mixed agents led to a better reflection of clinical practice and less selection bias. Despite the higher baseline BCVA score of 63.9 letters in our study population, resulting in a possible ceiling-effect, we observed a gain of +4.7 letters in eyes under TER at year 4 comparable to the improvements of +3.6 (7), respectively, +6.6 letters (8) after 4 years in similar populations. A meta-analysis of real-life studies using a TER with ranibizumab reported a BCVA gain of +5.4 after 3 years (4). In the present study 24.4% of all eyes gained more than 3 lines after 4 years which is only slightly below the 29.8% achieved in controlled clinical trials like the VIEW 1 extension study (19).

Concerning treatment intervals, our injection frequencies were higher than in other recent studies using aflibercept (7, 8) probably due to the stable proportion of 30% eyes requiring 4-week treatment intervals in our cohort. This might be explained by our good long-term treatment adherence and inclusion criteria namely allowing the switch between anti-VEGF agents, thus possibly including more poor responders. Accordingly, Mrejen et al. (18) report a similar number of injections per year in a cohort with a mean follow-up period of 3.5 years and mixed treatment agents (8.3 vs. 8.2, mean). While an increased duration of treatment effects using aflibercept has been described in eyes with limited response to ranibizumab, in our study the treatment distribution shifted from 90% ranibizumab in the first year toward 80% aflibercept in year 6 (20). However, due to the limited number of eyes treated with a single agent in our cohort, we did not analyze outcomes according to the administered drug.

In our study 30.3% of eyes reached the exit criteria whereas Jaggi et al. (8) report a similar proportion of 29% in a study with mean follow-up of 3.5 years with a comparable recurrence rate (34.4%/28% after 11.2 months/52.2 weeks, mean). Further, our visual acuity data after 5 years including eyes solely under observation compared to eyes still in TER were only slightly lower (68.5 vs. 71.1 letters), supporting the use of an exit strategy which results in good functional outcomes without unnecessary treatment burden (3).

We report a persistent reduction in CRT throughout the observation period with a stable proportion of a fluid-free OCT in around half of the eyes which supports previously published findings in smaller cohorts (7, 8). The integrity of the IS/OS interface and ELM improved with anti-VEGF treatment and stabilized in the long term, therefore supporting the short-term results of Oishi et al. (21) who showed the recovery of IS/OS and ELM in response to anti-VEGF therapy.

Type 1 lesions were reported to maintain better visual outcomes compared to the other phenotypes (18, 22, 23). Contrary to the results of Mrejen et al. (18) in our cohort type 2 lesions were correlated with higher BCVA gains compared to the other subtypes. However, these findings might partially be explained by their lower baseline BCVA as no significant association was shown in multivariate analysis. Yet, multivariate analysis identified a younger age and a worse baseline BCVA as predictors of better BCVA gain, findings previously described as a ceiling effect (18, 22, 24).

Our study is the first to analyze a multitude of OCT parameters as predictors for long-term visual outcomes in TER. The presence of central GA at baseline was associated with less long-term (5 years) BCVA gain. Accordingly, areas of hypoautofluorescence have been described as a predictor of poor anti-VEGF response at 1 year, additionally the visual potential in these patients is likely reduced due to the progression of the atrophic component (11, 22).

Photoreceptor integrity as evaluated by ELM and IS/OS interface has been shown as an important contributor to visual function in various macular pathologies (25, 26). In our study, a worse ELM disruption score at diagnosis predicted poor visual gain at 5 years which supports its previously described importance in predicting short-term visual acuity following the first injection (12) as well as after 6 (23) and 18 months (27), respectively. Since destruction of ELM reflects the impairment of photoreceptor nuclei and therefore severe retinal damage whereas IS/OS involves only the segments, ELM might serve as a better predictor of VA gain (11) even after the disappearance of the IS/OS interface. Likewise, Oishi et al. describe a higher correlation between BCVA and integrity of ELM than IS/OS in nAMD following photodynamic therapy, resulting from its higher sensitivity in advanced retinal damage (28).

The presence of a central PED has been associated with poor short-term visual outcome (22), increased recurrences (23) and poor response to anti-VEGF therapy at 1 year (29). In the VIEW 1 extension study, the vision loss after shifting to a PRN regimen was mostly attributed to recurrent fluid in eyes with PED at diagnosis (30). In a large retrospective study, the presence of a PED at treatment discontinuation after fulfilling exit criteria was a risk factor of recurrent disease (3). Whereas, in our study the presence of a central PED at baseline or at exit was not associated with an increased risk of recurrence, it was a significant predictor of VA gain at 1 year and the probability of reaching the exit criteria. Accordingly, tissue beneath the retinal pigment epithelium might impair both oxygen diffusion via the choroid and drug penetration from the vitreous.

Whereas, no previous studies investigated predictors of reaching exit criteria, Haddad et al. (31) suggest a significant increase in the incidence of yearly remissions with the years of anti-VEGF therapy using a PRN regimen. In our cohort a low baseline CRT and ELM integrity were associated with reaching the exit of TER within 5 years.

Limitations of this study lie in its retrospective design, hence including eyes at different time points between 2012 and 2020, therefore, the availability of anti-VEGF agents and the treat-and-extend protocol differed slightly. Analyzing both eyes in 24 patients, some interdependence between the eyes cannot be excluded. The high retention rate of 64% after 5 years with death as the main reason for a drop-out, the overall size of the study population as well as the follow-up of up to 8 years contribute to the strengths of our study. The inclusion of a population treated with mixed agents results in decreased selection bias. The treatment at a single department with subsequent follow-up even after reaching the exit criteria led to standardized disease management and enabled early recognition of recurrences.

To conclude, we report sustained long-term visual gains in a large cohort of eyes with treatment-naïve nAMD using a TER with exit-strategy exhibiting a great treatment adherence followed for up to 8 years. Baseline characteristics such as the integrity of ELM predicted the probability of reaching exit criteria within 5 years as well as long-term visual outcome and may be useful in adapting treatment strategies and handle patients' expectations.

## Data Availability Statement

The raw data supporting the conclusions of this article will be made available by the authors, without undue reservation.

## Ethics Statement

The studies involving human participants were reviewed and approved by Ethikkommission Nordwestschweiz, Switzerland. The patients/participants provided their written informed consent to participate in this study.

## Author Contributions

LH: acquisition and interpretation of data, statistics, and writing of manuscript. KH: design of the study, supervision, and writing of manuscript. All authors contributed to the article and approved the submitted version.

## Conflict of Interest

The authors declare that the research was conducted in the absence of any commercial or financial relationships that could be construed as a potential conflict of interest.

## Publisher's Note

All claims expressed in this article are solely those of the authors and do not necessarily represent those of their affiliated organizations, or those of the publisher, the editors and the reviewers. Any product that may be evaluated in this article, or claim that may be made by its manufacturer, is not guaranteed or endorsed by the publisher.

## References

[1] Brown DM Kaiser PK Michels M Soubrane G Heier JS Kim RY. Ranibizumab versus verteporfin for neovascular age-related macular degeneration. N Engl J Med. (2006) 355:1432–44. 10.1056/NEJMoa062655 17021319

[2] Heier JS Brown DM Chong V Korobelnik JF Kaiser PK Nguyen QD. Intravitreal aflibercept (VEGF trap-eye) in wet age-related macular degeneration. Ophthalmology. (2012) 120:209–10. 10.1016/j.ophtha.2012.09.006 23084240

[3] Arendt P Yu S Munk MR Ebneter A Wolf S Zinkernagel MS. Exit strategy in a treat-and-extend regimen for exudative age-related macular degeneration. Retina. (2019) 39:27–33. 10.1097/IAE.0000000000001923 29135888 PMC6325772

[4] Kim LN Mehta H Barthelmes D Nguyen V Gillies MC. Metaanalysis of real-world outcomes of intravitreal ranibizumab for the treatment of neovascular age-related macular degeneration. Retina. (2016) 36:1418–31. 10.1097/IAE.0000000000001142 27388744

[5] Hatz K Prünte C. Treat and Extend versus Pro Re Nata regimens of ranibizumab in neovascular age-related macular degeneration: a comparative 12 Month study. Acta Ophthalmol. (2017) 95:e67–e72. 10.1111/aos.13031 27009503

[6] Comparison of Age-related Macular Degeneration Treatments Trials (CATT) Research Group Maguire MG Martin DF Ying GS Jaffe GJ Daniel E . Five-year outcomes with anti-vascular endothelial growth factor treatment of neovascular age-related macular degeneration: the comparison of age-related macular degeneration treatments trials. Ophthalmology. (2016) 123:1751–61. 10.1016/j.ophtha.2016.03.045 27156698 PMC4958614

[7] Traine PG Pfister IB Zandi S Spindler J Garweg JG. Long-term outcome of intravitreal aflibercept treatment for neovascular age-related macular degeneration using a treat-and-extend regimen. Ophthalmol Retin. (2019) 3:393–9. 10.1016/j.oret.2019.01.018 31044729

[8] Jaggi D Nagamany T Ebneter A Munk M Wolf S Zinkernagel M. Aflibercept for age-related macular degeneration: 4-year outcomes of a ‘treat-and-extend' regimen with exit-strategy. Br J Ophthalmol. (2020) bjophthalmol-2020-316514. 10.1136/bjophthalmol-2020-316514 PMC8788035 33127830

[9] Berg K Roald AB Navaratnam J Bragadóttir R. An 8-year follow-up of anti-vascular endothelial growth factor treatment with a treat-and-extend modality for neovascular age-related macular degeneration. Acta Ophthalmol. (2017) 95:796–802. 10.1111/aos.13522 28926190

[10] Jaki Mekjavic P Zaletel Benda P. Outcome of 5-year treatment of neovascular age-related macular degeneration with intravitreal anti-VEGF using Treat and Extend regimen. Front Med. (2018) 5:125. 10.3389/fmed.2018.00125 29765959 PMC5938349

[11] Shin HJ Chung H Kim HC. Association between foveal microstructure and visual outcome in age-related macular degeneration. Retina. (2011) 31:1627–36. 10.1097/IAE.0b013e31820d3d01 21606888

[12] Zandi S Weisskopf F Garweg JG Pfister IB Pruente C Sutter F. Pre-existing RPE atrophy and defects in the external limiting membrane predict early poor visual response to ranibizumab in neovascular age-related macular degeneration. Ophthalmic Surg Lasers Imaging Retina. (2017) 48:326–32. 10.3928/23258160-20170329-07 28419398

[13] Hatz K Prünte C. Changing from a pro re nata treatment regimen to a treat and extend regimen with ranibizumab in neovascular age-related macular degeneration. Br J Ophthalmol. (2016) 100:1341–5. 10.1136/bjophthalmol-2015-307299 26755642

[14] Hatz K Ebneter A Tuerksever C Pruente C Zinkernagel M. Repeated dexamethasone intravitreal implant for the treatment of diabetic macular oedema unresponsive to Anti-VEGF Therapy: outcome and predictive SD-OCT features. Ophthalmologica. (2018) 239:205–14. 10.1159/000485852 29402873 PMC6008872

[15] Giannakaki-Zimmermann H Behrndt A Hoffmann L Guichard MM Türksever C Prünte C. Predictors for 2-year functional and morphological outcomes of a treat-and-extend regimen with ranibizumab in patients with diabetic macular edema. Ophthalmic Res. (2021) 64:465–75. 10.1159/000514721 33498045 PMC8259065

[16] Gregori NZ Feuer W Rosenfeld PJ. Novel method for analyzing snellen visual acuity measurements. Retina. (2010) 30:1046–50. 10.1097/IAE.0b013e3181d87e04 20559157

[17] Khanani AM Gahn GM Koci MM Dang JM Brown SM Hill LF. Five-year outcomes of intravitreal drug therapy for neovascular age-related macular degeneration in eyes with baseline vision 20/60 or better. Clin Ophthalmol. (2019) 13:347–51. 10.2147/OPTH.S191170 30858684 PMC6387604

[18] Mrejen S Jung JJ Chen C Patel SN Gallego-Pinazo R Yannuzzi N. Long-term visual outcomes for a treat and extend anti-vascular endothelial growth factor regimen in eyes with neovascular age-related macular degeneration. J Clin Med. (2015) 4:1380–402. 10.3390/jcm4071380 26239682 PMC4519796

[19] Kaiser PK Singer M Tolentino M Vitti R Erickson K Saroj N. Long-term safety and visual outcome of intravitreal aflibercept in neovascular age-related macular degeneration: VIEW 1 extension study. Ophthalmol Retina. (2017) 1:304–13. 10.1016/j.oret.2017.01.004 31047516

[20] Hatz K Prünte C. Intravitreal aflibercept in neovascular age-related macular degeneration with limited response to ranibizumab: a treat-and-extend Trial. Retina. (2017) 37:1185–92. 10.1097/IAE.0000000000001318 27652915

[21] Oishi A Shimozono M Mandai M Hata M Nishida A Kurimoto Y. Recovery of photoreceptor outer segments after anti-VEGF therapy for age-related macular degeneration. Graefes Arch Clin Exp Ophthalmol. (2013) 251:435–40. 10.1007/s00417-012-2034-4 22576370

[22] Ying GS Huang J Maguire MG Jaffe GJ Grunwald JE Toth C. Baseline predictors for one-year visual outcomes with ranibizumab or bevacizumab for neovascular age-related macular degeneration. Ophthalmology. (2013) 120:122–9. 10.1016/j.ophtha.2012.07.042 23047002 PMC3536921

[23] Ashraf M Souka A Adelman RA. Age-related macular degeneration: using morphological predictors to modify current treatment protocols. Acta Ophthalmol. (2018):120–33. 10.1111/aos.13565 29130626

[24] Zhang X Lai T. Baseline predictors of visual acuity outcome in patients with wet age-related macular degeneration. Biomed Res Int. (2018) 2018:9640131. 10.1155/2018/9640131 29682574 PMC5846359

[25] Eandi CM Chung JE Cardillo-Piccolino F Spaide RF. Optical coherence tomography in unilateral resolved central serous chorioretinopathy. Retina. (2005) 25:417–21. 10.1097/00006982-200506000-00004 15933586

[26] Yamaike N Tsujikawa A Ota M Sakamoto A Kotera Y Kita M. Three-dimensional imaging of cystoid macular edema in retinal vein occlusion. Ophthalmology. (2008) 115:355–62.e2. 10.1016/j.ophtha.2007.04.052 17675242

[27] Coscas F Coscas G Lupidi M Dirani A Srour M Semoun O. Restoration of outer retinal layers after aflibercept therapy in exudative AMD: prognostic value. Invest Ophthalmol Vis Sci. (2015) 56:4129–34. 10.1167/iovs.15-16735 26114491

[28] Oishi A Hata M Shimozono M Mandai M Nishida A Kurimoto Y. The significance of external limiting membrane status for visual acuity in age-related macular degeneration. Am J Ophthalmol. (2010) 150:27–32.e1. 10.1016/j.ajo.2010.02.012 20609705

[29] Suzuki M Nagai N Izumi-Nagai K Shinoda H Koto T Uchida A. Predictive factors for non-response to intravitreal ranibizumab treatment in age-related macular degeneration. Br J Ophthalmol. (2014) 98:1186–91. 10.1136/bjophthalmol-2013-304670 24711658 PMC4145467

[30] Schmidt-Erfurth U Waldstein SM Deak GG Kundi M Simader C. Pigment epithelial detachment followed by retinal cystoid degeneration leads to vision loss in treatment of neovascular age-related macular degeneration. Ophthalmology. (2015) 122:822–32. 10.1016/j.ophtha.2014.11.017 25578255

[31] Haddad WM Minous FL Legeai J Souied EH. Long-term outcomes and incidence of recurrence of neovascularization in treated exudative age-related macular degeneration. Retina. (2017) 37:951–61. 10.1097/IAE.0000000000001282 27617541

